# Test–retest reliability and practice effect of the Leuven Perceptual Organisation Screening Test

**DOI:** 10.3758/s13428-021-01741-z

**Published:** 2022-01-11

**Authors:** Xiaotong Ding, Kathleen Vancleef

**Affiliations:** 1 Present Address: Department of Experimental Psychology, University of Oxford, Anna Watts Building, Radcliffe Observatory Quarter, Woodstock Road, Oxford, OX2 6GG UK; 2 Department of Psychology, Durham University, DH1 3LE South Road, Durham, UK

**Keywords:** Visual assessment, test-retest reliability, Bland-Altman analysis, Practice effect, Intraclass correlation

## Abstract

Abstract

Visual diagnostic tests must have a high degree of consistency in their measurements (high reliability) to ensure accurate assessment of perceptual abilities. The current study assessed test–retest reliability and practice effects in the Leuven Perceptual Organisation Screening Test (L-POST) in 144 healthy volunteers, with time intervals between 0 and 756 days. We used Pearson's and intraclass correlation analysis, Bland–Altman analysis and multilevel modelling. Results from our analyses converged and supported an adequate reliability of the L-POST. Multilevel modelling demonstrated an absence of practice effect, suggesting that the L-POST is suitable for repeat administration. This study suggests that the L-POST has adequate reliability and is suitable for repeat administration even at short intervals. This study provides the basis for a more systematic evaluation for neuropsychological assessments, which can lead to the development of more reliable assessment batteries.

## Introduction

Visual perception is the extraction and interpretation of visual cues from the environment. It underlies our ability to acquire object knowledge and to interact effectively with others. However, it is easily affected by brain damage and neurological disorders (e.g., James et al., 2003). Various tests have been developed to measure the perceptual abilities retained after brain damage, such as the Leuven Perceptual Organisation Screening Test (L-POST) (Torfs et al., 2014).

The L-POST is a short online screening test of perceptual organisation ability—the grouping of individual visual elements into a coherent whole. Perceptual organisation is distinct from sensory vision (e.g., eye movements) *and* high-level vision such as face or object recognition. The L-POST consists of 15 subtests, each with five items designed to cover a range of mid-level processing, such as figure-background separation, texture/contour integration, object grouping, and figure completion. The test does not rely on intact long-term memory, expressive language**,** or motor control. The L-POST is widely applicable and efficient in use (administering takes only 20–45 min) and can be followed up by more in-depth testing of specific visual functions. The test is freely available at www.gestaltrevision.be/tests and is described in detail in Torfs et al. (2014).

To ensure accurate assessment of perceptual abilities, diagnostic tests must have high reliability—a high degree of consistency in its measurements (Cohen & Swerdlik, 2018). The reliability of a study can be assessed in many ways. For example, test–retest reliability refers to the consistency of measurements taken at two separate times (Cohen & Swerdlik, 2018). Commonly, Pearson’s correlation of the scores on two occasions is used as an index of the strength of the linear relationship between the variables. Adequate reliability is represented by a correlation between 0.7 and 0.8 (Taylor, 1990). Vancleef’s (2015) study has demonstrated an adequate test–retest reliability of the L-POST (*r* = .77, *p* < .001) using a sample of 20 stroke patients. However, test–retest correlations for the subtests varied widely, and about half were not significantly different from zero, suggesting large confidence intervals around the observed correlations. Because samples vary randomly, it is plausible that the obtained correlation is much larger or smaller than the true population correlation. The smaller the sample size, the greater the likelihood of obtaining a spuriously small correlation coefficient. It is therefore uncertain if the low correlations reflect a genuine poor test–retest reliability or are extreme estimates due to their small sample size. Schonbrodt and Perugini (2013) simulated the effect of sample sizes on correlation estimates. They recommend a minimum sample size of 129 participants to find at least 95% of the sample correlations between values of 0.6 and 0.8 given a true population correlation of 0.7. This suggests that the previous study on test–retest reliability of L-POST was underpowered.

Additionally, correlation analysis examines the relatedness rather than agreement between two measurements. For instance, if all participants made a similar improvement in the retest, there would be low agreement between test and retest, but the correlation coefficient would be high. Notably, such a practice effect is not uncommon in neuropsychological assessments (Calamia et al., 2012). Therefore, methods sensitive to the differences between measurements, such as the Bland–Altman analysis, are more preferable to evaluate test–retest reliability than a correlation analysis. Bland–Altman analysis reveals the discrepancy between two measurements by plotting their difference against their mean value for each corresponding pair of values (Bland & Altman, 2010). This method is also less affected by the range of values the variables take compared to correlation analysis (Karlijn et al., 2008). Bland–Altman analysis is therefore more appropriate than correlation analysis in the case of L-POST subtests, where the range is limited to five items per subtests. Similarly, methods accounting for the differences in scores for each person, such as the intraclass correlation coefficient, are more suited than Pearson’s correlations as a mean of assessing stability (Koo & Li, 2016).

Another problem associated with the traditional way of testing reliability is that comparing two sessions with a fixed time interval in between does not accurately represent the clinical reality. In a clinical context, an instrument is likely to be repeated more than once (e.g., to check progress over time when delivering intervention/treatment) and rarely ever at the time interval used in traditional reliability studies. It is therefore important to evaluate test–retest reliability at multiple time intervals and across multiple sessions to increase the ecological validity of a reliability study. Multilevel modelling allows for comparison of more than two sessions and the inclusion of additional predictors such as the time between test sessions (discrepancy between measures might be smaller at shorter time intervals than at longer time intervals) and the number of previous sessions (discrepancy between measures might be smaller with more practice with the test), while at the same time controlling for differences in baseline performance between individuals. Together, multilevel modelling and Bland–Altman analyses allow an in-depth examination of any practice effect if one exists.

The current study aims to evaluate test–retest correlations of the L-POST in a sufficiently large sample. Additionally, we evaluate practice effects through Bland–Altman analyses and multilevel modelling.

## Method

Our convenience sample consisted of 144 healthy volunteers (39 male, 105 female). Participants’ age ranged between 18.34 and 83.48 years (median = 23.28, interquartile range = 19.92–35.62). The mean time spent in education was 14.36 years (SD = 4.62). Participants’ country of residence was as follows: Belgium (58), Hungary (24), Italy (11), Turkey (10), Germany (9), United States of America (9), Taiwan, People’s Republic of China (6), Israel (3), Netherlands (3), Slovakia (2), United Kingdom (2), Australia (1), Aland Islands (1), Switzerland (1), Spain (1), France (1), Portugal (1), and Singapore (1). Most participants reported normal (66) or corrected-to-normal (75) vision. Three participants reported having an eye condition, but no further details were provided. None of the participants reported having any neurological disorders. All procedures were approved by the Commission for Medical Ethics of the University of Leuven (ML8800).

The L-POST is a free online test for mid-level visual perception available at https://psytests.be/clinicians/ and is described in detail in Torfs et al. (2014). In 15 subtests of five items each, participants choose one stimulus out of three that best resembles the target stimulus shown at the top of the screen. The validity and the internal structure of the L-POST were evaluated in detail in Vancleef et al. (2015). The study demonstrates convergent validity: moderate but significant correlations were observed between the total score on the L-POST and related tests of visual perception (e.g., Birmingham Object Recognition Battery (BORB), Riddoch & Humphreys, 1993; Rey Complex Figure Test, Meyers & Meyers, 1996; Visual Object and Space Perception battery (VOSP), Warrington & James, 1991). Additionally, small correlations between neuropsychological measures of other functions (e.g., spatial attention, executive functions, memory, language, number skills, and praxis) and performance on the L-POST indicates that the L-POST is specific for visual problems and that its performance is not highly influenced by other cognitive impairments, suggesting high discriminant validity. Confirmatory factor analyses indicates good fit indices for the theoretically implied structure of the L-POST based on the perceptual processes model: perceptual grouping, figure-ground segmentation, parts in wholes, and shape discrimination.

Participants completed the L-POST at least twice and up to 18 times in their preferred language (eight options available) on their own device (large enough screen for simultaneous presentation of all stimuli) at their preferred time and location. Sessions where participants reported a high level of interruptions or technical issues were excluded from the analyses. We included 320 sessions of our 144 participants: two sessions from 132 participants, three sessions from nine participants, six sessions from one participant, 10 sessions from one participant, and 13 sessions from one participant. The duration between sessions ranged between 4 minutes and 756 days, with a median of 16 days (interquartile range = 0–104 days).

Reliability was evaluated by comparing performance on the first and second session for which data were available (*N* = 144). The duration between the first and second sessions has a median of 26 days (range = 6 minutes to 756 days). Comparisons were made through test–retest correlations, Bland–Altman analysis and intraclass correlations (ICC). We calculated Pearson’s correlation for the continuous variable ‘total L-POST score’. Correlation coefficients exceeding 0.70 would be considered a demonstration of acceptable reliability (Nunnally & Bernstein, 1994). At the level of subtests, polychoric correlations were calculated to account for the limited range of possible values (0–5) and expected ceiling effects in most subtests (Vancleef et al., 2015). Permutation tests were used to calculate *p*-values. An acceptable level of agreement for the Bland–Altman analysis was set as one-third of the range of scores: 1.5 for the subtests (1/3 of 0–5) and 23 for the total score (1/3 of 0–70). For calculation of the ICC estimates and 95% confidence intervals, we used a two-way single-measurement mixed model with absolute agreement between scores of both sessions and under the expectation that a score of one session would generalise to other sessions. We followed Koo and Li’s (2016) recommendations for interpreting ICC values: values less than 0.5 are indicative of poor reliability, values between 0.5 and 0.75 indicate moderate reliability, values between 0.75 and 0.9 indicate good reliability, and values greater than 0.90 indicate excellent reliability.

The practice effect was explored using multilevel modelling using restricted maximum likelihood estimates with trial number and time between sessions as fixed effects and a random intercept to account for inter-individual differences in baseline performance. All analyses were performed using the statistical software R (R Development Core Team, 2020) using the packages ‘irr’ (Gamer et al., 2019), ‘blandr’ (Datta, 2018), ‘polycor’ (Fox, 2021), ‘nlme’ (Pinheiro et al., 2021), ‘MuMIn’ (Barton, 2020), ‘lmerTest’ (Kuznetsova et al., 2017), ‘ggplot2’ (Wickham et al., 2021) and ‘moments’ (Komsta & Novomestky, 2015). All analysis codes and data are made available to the readers [Data: 10.6084/m9.figshare.12789272, Analysis code: 10.6084/m9.figshare.12789281].

## Results

**Fig. 1:**
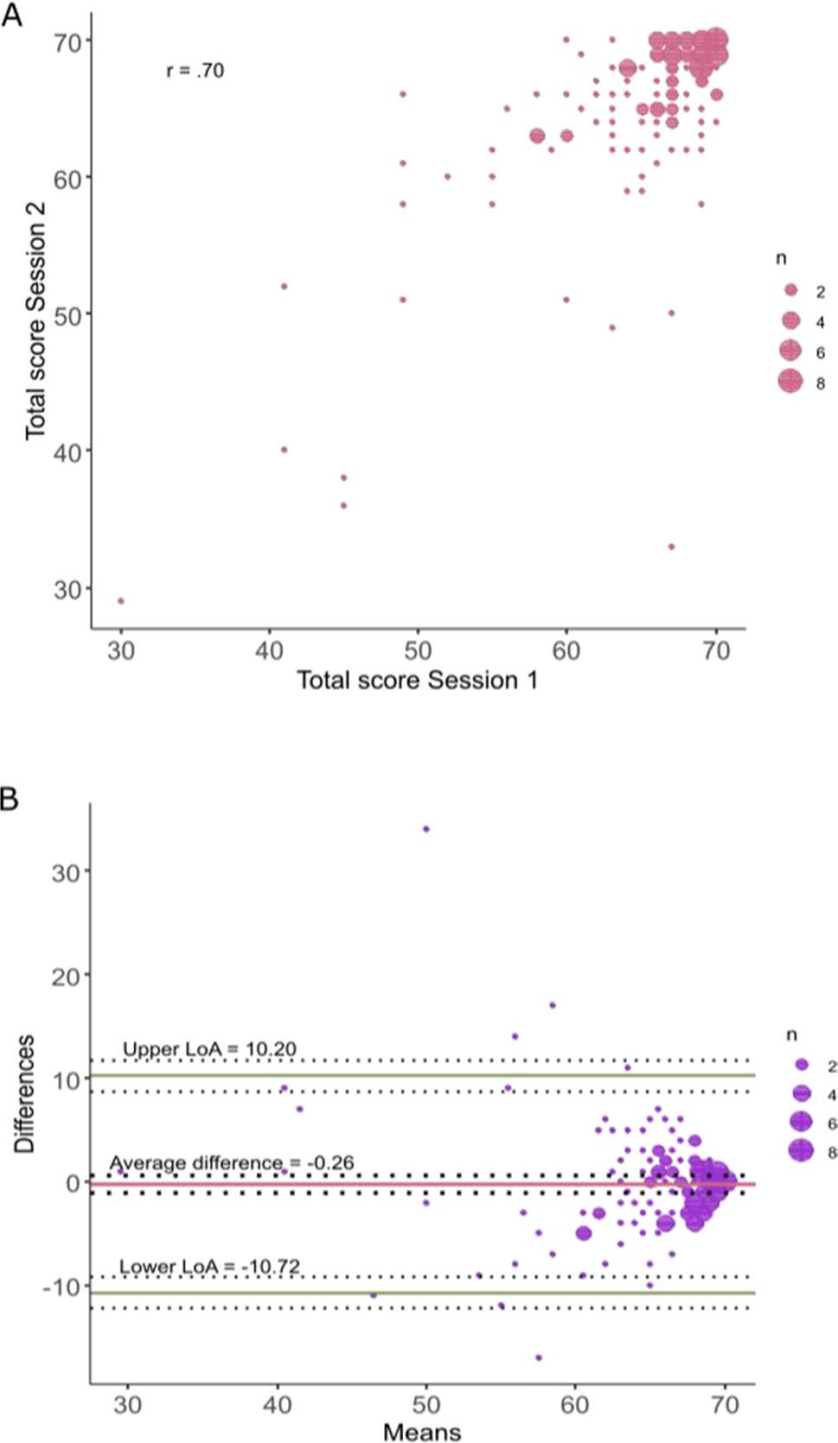
**a** A scatter plot depicting the correlation between total scores in session 1 and session 2. Pearson’s correlation coefficient *r* is presented at the top left corner of the graph (*r* = .70). The size of the dots reflects the number of overlapping points, with bigger dots representing more measurements. **b** A Bland–Altman plot comparing the means and differences in total scores in session 1 and session 2. The central red line represents the average difference in total scores across the two sessions (average difference = −0.26). The limits of agreement (LoA) are indicated by the green lines at the top and bottom of the graph (upper LoA = 10.20, lower LoA = −10.72). 95% confidence intervals for the average difference, the upper LoA, and the lower LoA are shown asdotted lines above and below each of the three lines. The size of the dots reflects the number of overlapping points, with bigger dots representing more measurements

The Pearson’s correlation of the total scores of the L-POST demonstrated adequate test–retest reliability (*r* = .70, *p* < .001, Fig. 1a). Polychoric correlations of subtests ranged between 0.22 and 0.79 and were significantly different from zero for all but two subtests (Table 1).

**Table.1:** Test–retest correlation and Bland–Altman analysis for each subtest

	Vancleef et al. (2015)	Current study			
Total score/subtests	Test–retest reliability (*n* = 20)	Test–retest reliability (*n* = 144)	Bland–Altman analysis		Intraclass correlation coefficient
			Mean difference	Upper and lower LoA	
Total score	**.77 (< .01)**	**.70 (< .001)**	-0.26 [-1.14, 0.62]	10.20, −10.72	0.70 [0.61, 0.77]
1. Fine shape discrimination	.53 (.07)	**.38 (< .001)**	0.07 [−0.09, 0.23]	1.93, −1.79	0.31 [0.15, 0.45]
2. Shape ratio discrimination (Efron)	.56 (.16)	.22 (.17)	0.03 [−0.08, 0.15]	1.45, −1.38	0.20 [0.04, 0.35]
3. Dot lattices	**.54 (.04)**	**.41 (< .001)**	−0.06 [−0.22, 0.11]	1.92, −2.03	0.26 [0.10, 0.40]
4. RFP fragmented outline	−.09 (.82)	**.67 (< .001)**	−0.05 [−0.15, 0.05]	1.14, −1.24	0.37 [0.22, 0.50]
5. RFP contour integration	.46 (.06)	**.59 (< .001)**	0.03 [−0.11, 0.17]	1.70, −1.64	0.54 [0.41, 0.65]
6. RFP texture surface	**.67 (.02)**	**.61 (< .001)**	−0.01 [−0.14, 0.13]	1.57, −1.59	0.53 [0.40, 0.64]
7. Global motion detection	**.94 (< .01)**	**.79 (< .001)**	−0.09 [−0.19, 0.01]	1.09, −1.27	0.71 [0.62, 0.78]
8. Kinetic object segmentation	**.77 (< .01)**	**.77 (< .001)**	0.02 [−0.06, 0.10]	0.96, −0.92	0.73 [0.64, 0.80]
9. Biological motion	**.51 (.04)**	**.64 (< .001)**	−0.22 [−0.40, −0.03]	2.03, −2.46	0.53 [0.40, 0.64]
10. Dot counting	**.56 (.03)**	**.55 (< .001)**	0.19 [0.03, 0.35]	2.11, −1.74	0.45 [0.32, 0.58]
11. Figure-ground segmentation	**.60 (< .01)**	**.55 (< .001)**	−0.01 [−0.15, 0.13]	1.67, −1.69	0.43 [0.29, 0.56]
12. Embedded figure detection	−.03 (.98)	**.53 (< .001)**	−0.06 [−0.24, 0.13]	2.17, −2.28	0.40 [0.26, 0.53]
13. Recognition of missing part	.50 (.08)	**.45 (< .001)**	−0.06 [−0.22, 0.10]	1.82, −1.95	0.30 [0.14, 0.44]
14–15. Object recognition in a scene	**.90 (< .01)**	.28 (.17)	−0.06 [−0.15, 0.03]	1.01, −1.13	0.32 [0.17, 0.46]

A bold value indicates a test–retest correlation that is significantly different from 0

The *p*-values of the polychoric correlations are shown between brackets

The 95% confidence intervals for mean differences in Bland–Altman analysis and for the ICC estimates are shown between square brackets

Bland–Altman analyses showed a mean difference between test and retest total scores of −0.26 (SD = 5.34). The 95% confidence interval (CI) for the mean difference was −1.14 to 0.62. The upper and lower limits of agreement (LoA) for total scores were 10.20 (95% CI [8.69, 11.70]) and –10.72 (95% CI [–12.23, –9.22]) respectively (Fig. 1b). The mean difference for subtests ranged from –0.22 to 0.19; limits of agreement lay between –2.46 and 2.17 (Table 1).

The estimated ICC for the total score was 0.7 with a 95% confidence interval of 0.61 to 0.77. This indicates ‘moderate’ to ‘good’ reliability of the total score. At the level of subtests, ICC estimates varied between 0.20 and 0.73, with relatively large confidence intervals, meaning that the reliability of the subtest scores ranged between ‘poor’ and ‘good’ reliability (Table 1).

**Fig. 2:**
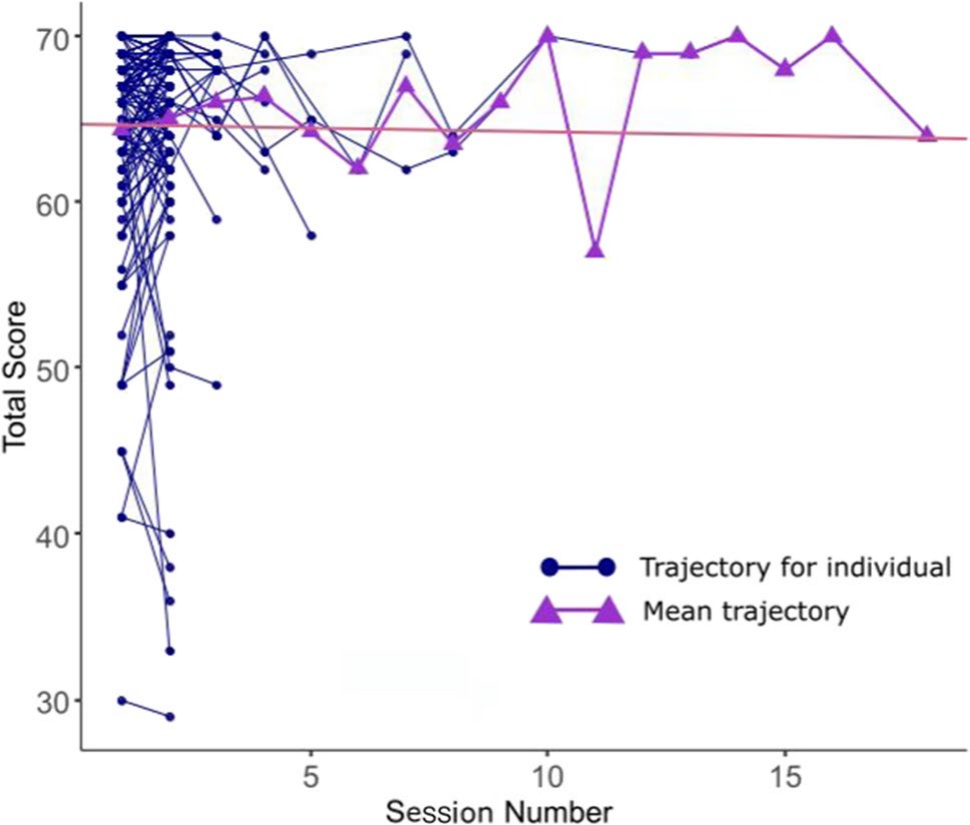
A spaghetti plot of total scores of 144 healthy volunteers at each L-POST session. Individual trajectories for total score are indicated by blue dots with a thin line connecting the total scores of multiple sessions for the same participant. Sessions where participants reported a high level of interruptions or technical issues were excluded from the analyses. For instance, session 17 of the one participant who completed 18sessions was excluded, and therefore no data are shown for session number 17. The bold purple triangles and connecting line illustrates the trajectory of average score across sessions. The red straight line represents the estimated regression line

Trajectories of changes in the individual scores over multiple sessions are presented in Figure 2. The multilevel model’s intercept (baseline performance) was estimated at 64.70 (*t*(173) = 109.85, *p* < .001). Within this model, the effect of session number was not significant (*β* = −0.045, *t*(173) *=* −0.31, *p* = .760), neither was the effect of time between sessions (*β* = 0.004, *t*(173) *=* 1.02, *p* = .308) or the interaction effect of session number and time between sessions (*β* = −0.000, *t*(173) *=* −0.16, *p* = .870). Despite the non-significant fixed effects, our multilevel model explained 70.60% of the variance of total score (conditional *R*^2^). Indeed, the variance explained by the fixed effects was only 0.26% (marginal *R*^2^), suggesting that most of the variance was explained by the random effect. In other words, it suggests high inter-individual variability in baseline scores and little effect of repeated testing and the time between sessions.

## Discussion

Test–retest correlation between the total scores of 0.70 suggests adequate reliability of the L-POST. This is in line with a previous report (Vancleef et al., 2015). Our coefficient also fits the range of test–retest correlations found in other visual assessments (Brown et al., 2010). At the subtest level, we found test–retest correlations between 0.22 and 0.79 that were significantly different from zero in all except two of the subtests, further supporting adequate reliability of the L-POST. However, the value of the correlation is small in some subtests (< 0.70), and reliability is therefore limited. ICC estimates also indicate adequate reliability for most subtests but poor reliability in certain subtests.

In contrast to previous work, the current study evaluated practice effects through Bland–Altman analyses and demonstrated good agreement between the measurements taken on two test occasions, supporting the conclusion from the correlation analyses. Furthermore, the results suggest that the reliability of L-POST is stable across all levels of visual perceptual skills and that a change of at least 10 points in the total score represents a genuine difference. However, for each subtest, limits of agreement fluctuated around ± 2, suggesting that any difference within two points may be due to measurement error. This can be problematic in the context of a five-point scale, where a two-point difference represents a 40% change in score.

Multilevel modelling confirmed the absence of a practice effect and showed that the time between sessions did not predict the total score. More importantly, our alternative approach addressed the lack of ecological validity that traditional reliability studies experience. We showed that test–retest reliability of L-POST is stable across different time intervals and for multiple sessions, which reflects the clinical reality better than a fixed time interval and only one retest. This suggests that the L-POST is suitable for repeat administration, even at short intervals.

A limitation of the current study is that ceiling effects in L-POST scores might have negatively affected the correlations. The high average level of education in the sample might also have resulted in higher scores. A previous study showed a significant but small effect of education levels on L-POST performance (*F*(4, 1565) = 26.01, *p* < .001, ω^2^ = .06). Despite a potential ceiling effect, our Bland–Altman analyses and multilevel modelling reached convergent results as the correlation analyses, supporting an adequate reliability of the L-POST. Future inclusion of patient populations would diversify the sample and minimise ceiling effects. Second, data were collected online with little control or knowledge of the test conditions. We compensated for this lack of control by using strict inclusion criteria: only data with no reported technical issues or interruptions were included in the analyses.

In conclusion, this study demonstrated adequate reliability of L-POST and the absence of a practice effect. The total score is sufficiently reliable, whereas meaningful interpretation of a change in subtest scores is only advisable when the difference is at least two points.

## Declarations

### Availability of data and materials (data transparency)

The data and materials for all experiments are available at 10.6084/m9.figshare.12789272.

### Code availability (software application or custom code)

The analysis codes that support the findings of this study are openly available in figshare at 10.6084/m9.figshare.12789281.
